
JOURNAL INFORMATION
==============================
NLM Title Abbreviation: Schizophr Bull
Journal ID: Schizophr Bull
NLM Journal ID: 0236760
Journal ID: schbul

Schizophrenia Bulletin

ISSN: 0586-7614
EISSN: 1745-1701
Publisher: Oxford University Press

ARTICLE INFORMATION
==============================
PMCID: PMC5887676
DOI: 10.1093/schbul/sby016.439
Article ID: sby016.439
Article version: 1
Subjects: Abstracts, Poster Session I

T163. Submission Withdrawn

Print publication date: 2018 Apr
Electronic publication date: 2018 Apr 1
Volume: 44
Issue: Suppl 1
Issue title: Abstracts for the Sixth Biennial SIRS Conference
First page: S179
Last page: S179
Copyright: © Maryland Psychiatric Research Center 2018.
Copyright year: 2018
License: This is an Open Access article distributed under the terms of the Creative Commons Attribution License (http://creativecommons.org/licenses/by/4.0/), which permits unrestricted reuse, distribution, and reproduction in any medium, provided the original work is properly cited.
License URL: https://creativecommons.org/licenses/by/4.0/

Page count: 1

==============================
### T163. SUBMISSION WITHDRAWN

### T164. STRUCTURAL COVARIANCE IN DRUG-NAÏVE FIRST EPISODE PSYCHOSIS: AN ULTRA-HIGH FIELD MRI STUDY

Tushar Das[1], Kara Dempster[2], Michael Mackinley[2], Peter Jeon[1], Joe Gati[3], Jean Theberge[4], Ali Khan[3], Lena Palaniyappan*[,1]

*[1]University of Western Ontario; [2]London Health Sciences Center; [3]Robarts Research Institute; [4]St. Joseph's Health Care London*

**Background:** Structural neuroimaging studies report disrupted morphological relationship in the grey matter volume (structural covariance) in patients with schizophrenia, indicating an impairment in functional and/or developmental plasticity. To our knowledge, no studies have examined the alterations in structural covariance across the entire brain in drug-naïve first episode psychosis. TOPSY (Tracking Outcomes of Psychosis) is one of the first studies intending to track the neurobiological trajectory using ultra-high field (7T) imaging starting from a drug-naïve first episode state. Here, we report the initial findings from the structural covariance of grey matter volume. To our knowledge, this is the first structural covariance analysis being reported using a 7T anatomical MRI acquisition.

**Methods:** We used ultra-high field (7 Tesla) MRI in 28 patients with FEP (satisfying criterion A of DSM-5 schizophrenia) and 18 controls, to estimate grey matter volume in a voxelwise manner. FEP and controls were matched for age, sex and parental socioeconomic status. Patients were recruited at an early intervention unit (PEPP, London Ontario) and had active psychotic symptoms at the time of scanning. Morphometric analysis was done using SPM12, after DARTEL based registration and segmentation but without spatial smoothing on 160 brain regions (6mm spheres) obtained using Dosenbach's atlas. Correlation matrix for each group was constructed from 160*160 pearson correlation coefficients, followed by estimation of a bias matrix for each subject using jack-knife bias estimation. Bias values for each pair of nodes in an individual subject quantified the contribution of that subject to the overall within-group covariance. Higher positive values meant greater covariance between the two given nodes in that subject, relative to the rest of the group. These bias matrices can be considered equivalent to demeaned and normalised matrices of structural covariance. Structural covariance across all possible regional pairwise connections was tested using 2-tailed voxelwise T-test with FDR correction (p=0.05, 5% rate for false positives).

**Results:** Patients had a significant reduction in structural covariance affecting between right posterior insula and right precentral gyrus (within sensorimotor network, t=3.86, Hedge's g = 1.15); between right posterior insula and left ventral prefrontal cortex (between sensorimotor and salience network, t=3.71, Hedge's g = 1.10); and between right anterior cingulate cortex and right dorsal prefrontal cortex (between sensorimotor and default-mode network, t=3.10, Hedge's g = 0.92). There were no pairwise connections with increased structural covariance among FEP subjects compared to healthy controls.

**Discussion:** Our findings suggest that (1) structural covariance is disrupted even by the time of first-episode of psychosis; thus, the disruptions in morphological relationships reported in schizophrenia are not explicable by antipsychotic usage or illness duration (2) sensorimotor network regions show a predominant disruption in structural covariance, affecting morphological relationships with both salience and default mode regions. The functional and developmental plasticity of sensorimotor networks may be crucial for the early trajectory of psychosis.

### T165. ULTRA-HIGH FIELD MORPHOMETRY IN DRUG-NAÏVE FIRST EPISODE PSYCHOSIS

Tushar Das[1], Kara Dempster[2], Michael Mackinley[2], Peter Jeon[1], Joe Gati[3], Jean Theberge[1], Ali Khan[3], Lena Palaniyappan*[,1]

*[1]University of Western Ontario; [2]London Health Sciences Center; [3]Robarts Research Institute*

**Background:** Structural neuroimaging studies report distributed grey matter volume (GMV) deficits in drug-naïve first episode psychosis (FEP), though their relevance to symptom burden and cognitive deficits is currently unclear. When compared to studies in medicated patients and/or patients with established later-stage of psychosis, the GMV deficits reported have been limited in both spatial distribution and effect size, indicating the possibility of stage-specific progression during the clinical course of psychosis. TOPSY (Tracking Outcomes of Psychosis) is one of the first studies intending to track the neurobiological trajectory using ultra-high field (7T) imaging starting from a drug-naïve first episode state. Here, we report the initial findings from the voxel-based morphometry (VBM) of GMV. To our knowledge, this is the first VBM report from drug-naïve FEP subjects obtained using a 7T MRI acquisition.

**Methods:** We used ultra-high field (7 Tesla) MRI in 28 patients with FEP (satisfying criterion A of DSM-5 schizophrenia) and 18 controls, to evaluate differences in the grey matter. Volume in a voxelwise manner. FEP and controls were matched for age, sex and parental socioeconomic status. Patients were recruited at an early intervention unit (PEPP, London Ontario) and had active psychotic symptoms at the time of scanning. We also obtained abbreviated PANSS (8 items) scores to index the severity of psychosis. Analysis was done using SPM12, after DARTEL based registration and segmentation but without spatial smoothing. 2-tailed voxelwise T-test with FDR correction (p=0.05, 5% rate for false positives) was used. We used multiple regression analysis to predict the scores from processing speed measure (modified Symbol Substitution Test) and the severity of Delusions and Unusual Thought Content (P1 and G9), the 2 symptoms for which most subjects sought treatment in the first place.
